# Estimating Young Adult Uptake of Smoking by Area Across the United Kingdom

**DOI:** 10.1093/ntr/ntae231

**Published:** 2024-10-25

**Authors:** Sarah E Jackson, Harry Tattan-Birch, Nicholas S Hopkinson, Jamie Brown, Lion Shahab, Laura Bunce, Anthony A Laverty, Deborah Arnott

**Affiliations:** Department of Behavioural Science and Health, University College London, London, UK; SPECTRUM Consortium, Edinburgh, UK; Department of Behavioural Science and Health, University College London, London, UK; SPECTRUM Consortium, Edinburgh, UK; National Heart and Lung Institute, Faculty of Medicine, Imperial College London, London, UK; Department of Behavioural Science and Health, University College London, London, UK; SPECTRUM Consortium, Edinburgh, UK; Department of Behavioural Science and Health, University College London, London, UK; SPECTRUM Consortium, Edinburgh, UK; Action on Smoking and Health, London, UK; School of Public Health, Imperial College London, London, UK; Action on Smoking and Health, London, UK

## Abstract

**Introduction:**

There is majority support in parliament and across the United Kingdom to implement a “smoke-free generation” policy which would mean people born on or after January 1, 2009, could never legally be sold tobacco. To explore the potential impact this policy could have, we estimated the number of young adults (18–25 years) currently taking up smoking each year by area across the United Kingdom.

**Methods:**

Using data from the Office for National Statistics (ONS), Annual Population Survey (APS), and Smoking Toolkit Study (STS), we estimated the total number of 18- to 25-year-olds taking up smoking each year, based on national estimates of population size (ONS) and the proportion who reported ever having regularly smoked (STS). We used local data on adult smoking rates (APS) to apportion the national estimated number of young adults taking up smoking to specific areas.

**Results:**

Around 127 500 18- to 25-year-olds in the United Kingdom start smoking regularly each year (~350 each day); 105 700 each year in England, 11 500 in Scotland, 6500 in Wales, and 3800 in Northern Ireland. Uptake estimates varied across localities: for example, North East Lincolnshire had the highest proportion of young adults taking up smoking each year (3.9%) and Wokingham had the lowest (0.9%).

**Conclusions:**

Despite reductions in smoking prevalence over recent decades, hundreds of young adults in the United Kingdom start smoking every day.

**Implications:**

Data on rates of uptake among individual local authorities can be used to focus attention locally prior to the introduction of new age of sale laws.

## Introduction

Tobacco smoking is uniquely harmful and remains a leading cause of disease, disability, and premature death globally.^1^ Two out of every three people who do not manage to quit will die from their smoking.^2^

Most people take up smoking when they are young,^3,4^ underestimating the short-term risks and not expecting it to become a life-long habit.^5^ However, they quickly become addicted^6^ and find it difficult to quit in later life. The majority (54%) of people who smoke in England want to stop^7^ and three-quarters say they would never have started if they had the choice again.^8^ Starting to smoke at any age has severe health consequences in the long run, but these are particularly pronounced among those who start young.^9^

To reduce the number of young adults taking up smoking and alleviate the burden of smoking-related death and disease for the next generation, the UK Government intended to implement a “smoke-free generation” policy which would increase the legal age of sale of tobacco from 18 by 1 year each year from 2027 onwards, such that people born on or after January 1, 2009, would never legally be sold tobacco.^10^ A bill to enact the legislation was progressing through parliament—and passed a second reading by a majority of 316^11^—but did not pass into law before a snap election was called in May 2024. The new Government has committed to bringing the legislation back.

To offer insight into the potential impact this policy could have, this study aimed to estimate the number of young adults (18–25 years) currently taking up smoking each year by local area across the United Kingdom.

## Methods

### Estimating the Number of 18- to 25-Year-Olds in the United Kingdom Taking up Smoking

The initial analysis was based on data from the Smoking Toolkit Study, a nationally representative monthly cross-sectional survey of adults (≥16 years) in Great Britain.^12^ We analyzed data from 7080 respondents aged 16–25 years surveyed between January 2022 and January 2024.

Smoking status was assessed by asking participants which of the following best applied to them: (a) I smoke cigarettes (including hand-rolled) every day; (b) I smoke cigarettes (including hand-rolled), but not every day; (c) I do not smoke cigarettes at all, but I do smoke tobacco of some kind (eg, pipe, cigar, or shisha); (d) I have stopped smoking completely in the last year; (e) I stopped smoking completely more than a year ago; (f) I have never been a smoker (ie, smoked for a year or more). Those who responded (a)–(e) were considered to have ever regularly smoked.

Weighted logistic regression was used to model ever-regular smoking by age, with age modeled nonlinearly using restricted cubic splines (with three knots placed at the 5th, 50th, and 95th percentiles). We used this model to estimate the proportion of 17- and 25-year-olds who have ever regularly smoked, incorporating information from all participants aged 16–25 (rather than just those aged 17 and 25).

As a simplifying assumption, we assumed a constant rate of uptake of smoking across each year of aging, which we calculated as:


$$\begin{array}{l} \begin{array}{llll} & (\;proportion\;of\;25\text{-}year\text{-}olds\;who\;have\;ever\;regularly\; & \;smoked-proportion\;of\;17\text{-}year\text{-}olds\;who\;have\; & ever\;regularly\;smoked)/8\;years=rate\;of\;uptake\; \end{array} \end{array}$$

Using 2021 Office for National Statistics midyear estimates for population size,^13^ we then estimated the number of 18- to 25-year-olds in the United Kingdom who start smoking regularly each year (assuming the rate of uptake is the same as the Smoking Toolkit Study estimate for Great Britain) as:


$$\begin{array}{l} \begin{array}{llll} & rate\;of\;uptake*number\;of\;18\text{-}\;to\;25\text{-}year\text{-}olds\; & =number\;of\;18\text{-}\;to\;25\text{-}year\text{-}olds\;taking\;up\; & \;smoking\;each\;year\; \end{array} \end{array}$$

### Estimating Geographic Differences in the Number of 18- to 25-Year-Olds Taking up Smoking

This UK estimate was then split across geographical areas according to their adult smoking prevalences, based on the assumption that there was likely to be a greater proportion of young adults taking up smoking in areas that have more adults who smoke (ie, regional differences in smoking uptake would be proportionate to regional differences in adult smoking prevalence).

We obtained data on adult (≥18 years) and young adult (18–25 years) population sizes and adult smoking prevalence in the United Kingdom, overall and by nation (England, Scotland, Wales, and Northern Ireland), region in England, and upper-tier local authority and unitary authority areas of England, Wales, Scotland, and Northern Ireland. We used the 2021 Office for National Statistics midyear estimates for population size^13^ and the 2022 Annual Population Survey estimates of smoking prevalence.^14^

For each locality, we calculated the number of adults who smoke (local adult population size × local smoking prevalence) and the proportion of UK adults who smoke that this represented (local number of adults who smoke/total number of adults who smoke in the United Kingdom). We then apportioned our estimate of the total UK number of young adults taking up smoking according to the proportion of the total adult smoking population in that locality. We calculated the local number (total number of young adults in the United Kingdom taking up smoking each year × local proportion of UK adults who smoke) and proportion (local number of young adults taking up smoking each year/local young adult population size) of young adults taking up smoking each year. This apportioning method has been used in previous work by Action on Smoking and Health.^4^

We rounded all percentages to one decimal place and numbers <100 to the nearest 5, ≥100 and <1000 to the nearest 10, ≥1000 and <1 000 000 to the nearest 100, and ≥1 000 000 to the nearest 1000.

The analyses were not preregistered and should be considered exploratory.

## Results

The Smoking Toolkit Study analysis suggested the prevalence of ever-regular smoking increases from 21.9% at 17 years to 37.7% by age 25. Assuming a constant rate of uptake, this equates to 2.0% uptake across any year of aging between ages 18 to 25 ([37.7%–21.9%]/8 years). There are ~6 375 000 people aged 18–25 in the United Kingdom.^13^ We therefore estimated that 127 500 (6 375 000 * 2.0%) 18- to 25-year-olds in the United Kingdom start smoking regularly each year.

**Figure 1.**
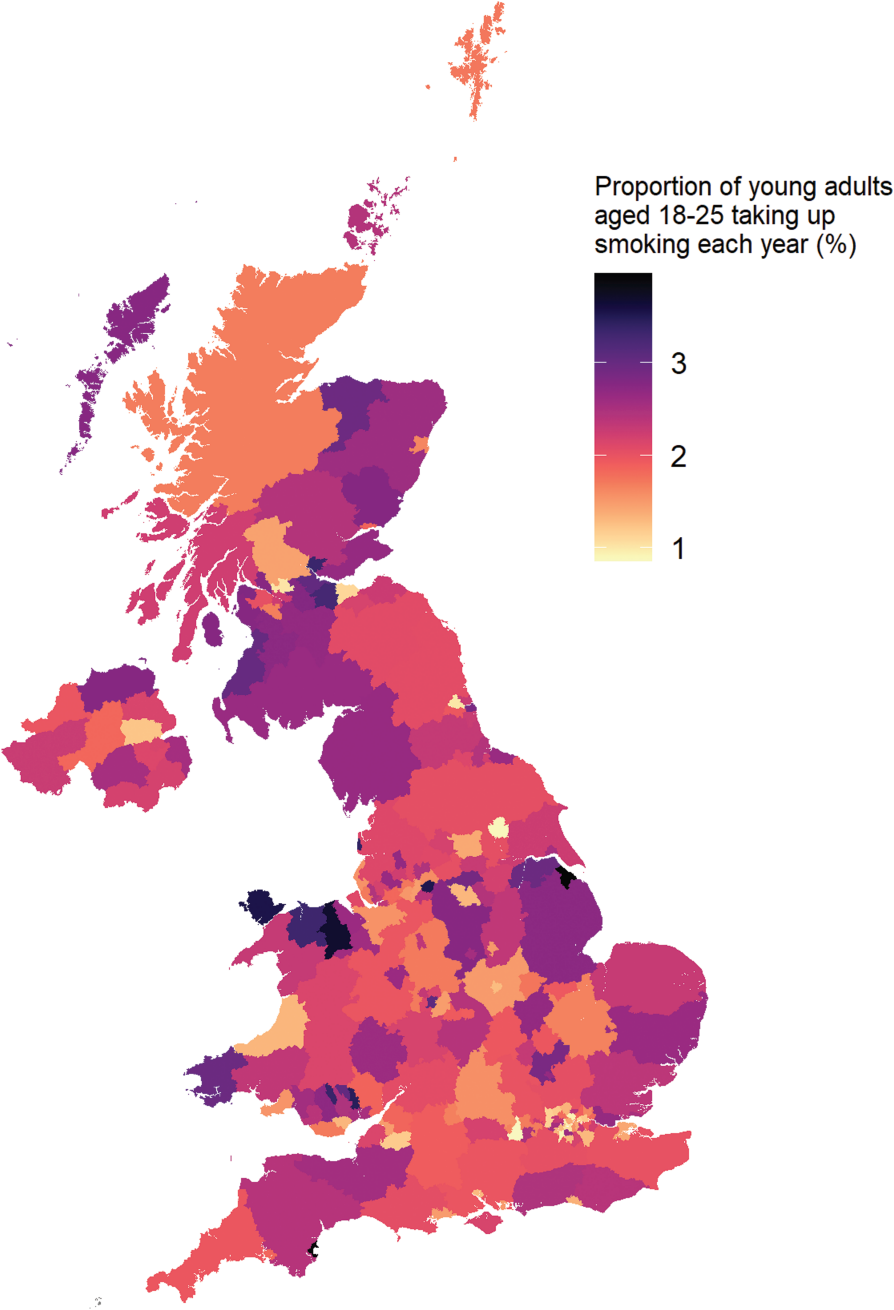
Estimated proportion of young adults aged 18–25 who start smoking in the United Kingdom each year. For each locality, data shown are the estimated total number of young adults taking up smoking in the United Kingdom each year apportioned according to the proportion of the total adult smoking population in that locality, divided by the local young adult population size.

The estimated proportion of 18- to 25-year-olds who take up smoking each year is presented as a heat map by local authority and unitary authority areas across the United Kingdom in Figure 1. Tables with daily, weekly, monthly, and annual figures for each UK nation and region in England are provided in Table 1; corresponding estimates for local authority and unitary authority areas are available in the Supplementary File.

**Table 1.** Estimates of Smoking Uptake Among 18- to 25-Year-Olds by UK Nation and Region in England

	Adult population^a^	Smoking prevalence, %^b^	No. of adults who smoke^c^	% of UK adult smoking population	Young adult population (18–25y)^1^	Number taking up smoking^d^ per…		
						Year	Month	Week
United Kingdom	53 188 000	12.9	6 861 000	100.0	6 375 000	127 500	10 600	2500
Nation								
England	44 775 000	12.7	5 686 000	82.9	5 392 000	105 700	8800	2000
Scotland	4 455 000	13.9	619 200	9.0	515 600	11 500	960	220
Wales	2 489 000	14.1	350 900	5.1	291 500	6500	540	130
Northern Ireland	1 470 000	14.0	205 800	3.0	176 600	3800	320	75
Region in England								
East Midlands	3 890 000	14.0	544 600	7.9	480 300	10 100	840	190
East England	5 018 000	13.2	662 400	9.7	546 200	12 300	1000	240
London	6 904 000	11.7	807 800	11.8	925 100	15 000	1300	290
North East	2 122 000	13.1	278 000	4.1	254 900	5200	430	100
North West	5 860 000	13.4	785 200	11.4	714 700	14 600	1200	280
South East	7 358 000	11.5	846 200	12.3	825 900	15 700	1300	300
South West	4 626 000	11.9	550 500	8.0	526 400	10 200	850	200
West Midlands	4 663 000	13.4	624 800	9.1	575 700	11 600	970	220
Yorkshire and the Humber	4 335 000	13.1	567 900	8.3	542 500	10 600	880	200

Estimated numbers taking up smoking by UK nation or region in England do not sum precisely to totals due to rounding.

^a^Office for National Statistics 2021 midyear population estimates.

^b^Annual Population Survey, 2022.

^c^Adult population × smoking prevalence.

^d^Estimated number of 18- to 25-year-olds taking up smoking.

Of the 6.375 million 18- to 25-year-olds across the United Kingdom, an estimated 127 500 start to smoke each year (~350 each day): 105 700 in England, 11 500 in Scotland, 6500 in Wales, and 3800 in Northern Ireland. There was substantial variation in the proportion of young adults taking up smoking across localities. North East Lincolnshire and Torbay had the highest rates of uptake (3.9% and 3.9% annually): each year, around 500 from a population of 12 700 in North East Lincolnshire and 390 from a population of 10 000 in Torbay started to smoke regularly. Uptake was lowest in Wokingham and York (0.9% and 0.9% annually), with around 120 and 270 young adults starting to smoke regularly each year from populations of 13 800 and 30 900, respectively.

## Discussion

This analysis shows that despite reductions in smoking prevalence over recent decades,^14^ hundreds of young adults in the United Kingdom start smoking every day. Raising the legal age of sale is likely to be an effective measure to reduce this, based on evidence that increases from 16 to 18 in the United Kingdom in 2007^15^ and increases to 21 in the United States^16,17^ were associated with decreases in smoking among age groups that could not legally able to buy tobacco. Steadily increasing the legal age of sale further will likely reduce the chance of people becoming addicted at any point in life, given older age of smoking initiation is associated with lower levels of lifetime nicotine dependence.^18^ As well as the overall scale of uptake, the data here on rates of uptake among individual local authorities can be used to focus attention locally on the value to their communities of the national policy to raise the age of sale to help prevent smoking initiation at any age.

Our analyses provide an estimate of the maximum possible number of young adults who, in theory, could be prevented from taking up smoking by the proposed smoke-free generation policy. However, it is likely that the real impact would be lower than this. Some people who are unable to legally be sold tobacco may find ways to circumvent the legislation, such as (legally) purchasing tobacco abroad and bringing it back to the United Kingdom or buying from the United Kingdom’s illicit market.^19^ The UK Government has implemented effective strategies that have seen the illicit market share of tobacco products fall considerably since 2000, and recently committed to significant additional investment in enforcement.^19^ Nonetheless, it is important to consider potential unintended consequences of the smoke-free generation policy (such as a growing illicit market), and strategies to mitigate them, to ensure the net effect is positive.

It is important to acknowledge that this type of estimation has limitations. The data presented are extrapolated from survey data, so are necessarily approximate. For some local and unitary authority areas, Annual Population Survey estimates of smoking prevalence were based on small samples so may be unreliable (for reference, sample sizes are provided in the Supplementary File). The Smoking Toolkit Study does not cover Northern Ireland, so the rate of uptake was estimated based on data collected in Great Britain, rather than the whole of the United Kingdom. As a simplifying assumption, we assumed a constant rate of uptake of smoking across years of aging between the ages of 17 and 25. However, our model suggested the rate of uptake was greater at younger ages within this age range, so estimates for each locality may be more or less accurate depending on the age distribution of the local young adult population. We also assumed that current population sizes were similar to 2021 and that local variation in rates of smoking uptake would correspond to variation in adult smoking rates.

## Supplementary Material

Supplementary material is available at *Nicotine and Tobacco Research* online.

ntae231_suppl_Supplementary_File

## Data Availability

Data and code are available on Open Science Framework (https://osf.io/nu2rp/).
